# Editorial Note: *In silico* analyses identify lncRNAs: WDFY3-AS2, BDNF-AS and AFAP1-AS1 as potential prognostic factors for patients with triple-negative breast tumors

**DOI:** 10.1371/journal.pone.0357419

**Published:** 2026-09-02

**Authors:** The PLOS One Editors

The *PLOS One* Editors issue this Editorial Note to inform readers that works cited in this article [1] as References 14, 33, 54, and 60 were retracted after the article’s publication date. PLOS considers that these references are not crucial in supporting the research or conclusions reported in [1], but the following sentences in the Discussion section are no longer supported:

- The sixth sentence of the second paragraph: In ovarian carcinoma, Li et al. identified reduced WDFY3-AS2 expression in tumor tissue compared to adjacent normal tissue.
- The fifth and sixth sentences of the fourth paragraph: In vitro assays, demonstrated that AFAP1-AS1 knockdown resulted in reduced proliferation and invasion and increased apoptosis rates in MDA-MB-231 and BT-549 cell lines. In vivo, overexpression of AFAP1-AS1 promoted tumor growth and activated Wnt/β-catenin pathway to promote tumorigenesis and cell invasion by increasing the expression of C-myc and EMT related genes.
